# Effects of daidzein on antioxidant capacity in weaned pigs and IPEC-J2 cells

**DOI:** 10.1016/j.aninu.2022.06.014

**Published:** 2022-07-14

**Authors:** Yanpin Li, Xianren Jiang, Long Cai, Yanli Zhang, Hongbiao Ding, Jingdong Yin, Xilong Li

**Affiliations:** a Key Laboratory of Feed Biotechnology of Ministry of Agriculture and Rural Affairs, Institute of Feed Research, Chinese Academy of Agricultural Sciences, Beijing 100081, China; b State Key Laboratory of Animal Nutrition, College of Animal Science and Technology, China Agricultural University, Beijing 100193, China

**Keywords:** Daidzein, Growth performance, Antioxidant capacity, IPEC-J2, Oxidative stress, Pig

## Abstract

Our previous study found that soybean isoflavones in soybean meal play an important role in improving growth performance and antioxidant capacity in pigs. However, it is still unknown whether long-term supplementation with daidzein, an active molecule deglycosylated from daidzin, in a corn–soybean meal diet can enhance growth performance in pigs. Thus, in the present study, an animal trial was carried out to investigate the effects of dietary supplementation with daidzein on the growth performance and antioxidant capacity of pigs. A total of 80 weaned piglets (40 barrows and 40 females) were assigned to 4 treatments with 5 pens per treatment and 4 piglets per pen and fed a diet supplemented with 0, 25, 50 and 100 mg/kg daidzein for a 72-day trial. In addition, porcine intestinal epithelial cells (IPEC-J2) were used as an in vitro model to explore the underlying antioxidant mechanisms of daidzein. IPEC-J2 cells were treated with 0.6 mM hydrogen peroxide (H_2_O_2_) in the presence or absence of 40 μM daidzein. The results showed that adding 50 mg/kg of daidzein to the diet significantly improved body weight on day 72, average daily gain (ADG) during days 0 to 72 and plasma superoxide dismutase (SOD) activity on day 42 (*P* < 0.05). Treatment with 0.6 mM H_2_O_2_ for 1 h significantly decreased cell viability and catalase (CAT) activity and increased intracellular reactive oxygen species (ROS) levels and malondialdehyde (MDA) content (*P* < 0.05), while pretreatment with 40 μM daidzein prevented the decrease in cell viability and CAT activity and the increase in intracellular ROS levels and MDA content caused by H_2_O_2_ (*P* < 0.05). In addition, H_2_O_2_ stimulation significantly suppressed the expression of nuclear factor erythroid-2-related factor 2 (*Nrf2*), *CAT*, occludin and zonula occludens-1 (*ZO-1*), while pretreatment with daidzein preserved the expression of *Nrf2, CAT* and occludin in H_2_O_2_-stimulated IPEC-J2 cells (*P* < 0.05). In conclusion, our results suggested that long-term dietary supplementation with 50 mg/kg daidzein improved growth performance in pigs and was beneficial to the antioxidant capacity of pigs. Daidzein exerted protective effects against H_2_O_2_-induced oxidative stress in IPEC-J2 cells and the underlying mechanism may be related to the activation of the Nrf2 signaling pathway.

## 1 Introduction

The antioxidant system of the body maintains a balance between the generation and elimination of reactive oxygen species (ROS). Oxidative stress occurs when the production of ROS exceeds the antioxidant capacity of the body (Qin et al., 2015). It damages DNA, proteins and lipids, eventually leading to diseases such as aging, cardiovascular disease and Alzheimer's disease (Dai and Rabinovitch, 2009; Sachidanandam et al., 2005; Owen et al., 1997). Oxidative stress in the intestinal tract can injure the intestinal structure, increase the permeability of epithelial cells and influence the absorption capability, eventually inducing gastrointestinal diseases, such as inflammation and cancer (Circu and Aw, 2012). Protecting intestinal cells from the damage caused by oxidative stress will preserve intestinal function, thereby improving growth performance in pigs.

Our previous study showed that eliminating soybean isoflavones from the diet decreased body weight (BW) and antioxidant capacity, while replenishing soybean isoflavones prevented a decrease in BW and antioxidant capacity (Li et al., 2020), indicating that soybean isoflavones are beneficial for pig growth and play an essential role in antioxidation. Daidzin and genistin are 2 major components of soybean isoflavones. Soybean isoflavones are deglycosylated to aglycones and absorbed by the intestine (Cools et al., 2014; Walsh et al., 2009). Daidzein (4,7-dihydroxyisoflavone) is deglycosylated from daidzin. The effects of daidzein on the growth of pigs have been reported in previous studies. Adding 200 mg/kg of daidzein to a diet without any soy source for 70 days or increasing dietary soybean meal level from 17.5% to 29% for 28 days did not affect pig growth (Xiao et al., 2015; Rochell et al., 2015). In addition, the antioxidant capacity of daidzein has been demonstrated in several studies. Adding 200 mg/kg of daidzein to a diet without any soy source significantly improved the antioxidant capacity of pigs (Xiao et al., 2015). In in vitro studies, the protective effects of daidzein against oxidative stress in human umbilical vein endothelial cells (HUVECs) and Caco-2 cells were evaluated (Xu et al., 2009; Wijeratne and Cuppett, 2007). Nevertheless, there is limited literature concerning whether long-term supplementation with daidzein in corn–soybean meal diets affects the growth performance of weaned and growing pigs, and whether daidzein exerts antioxidant capacity or protective effects against oxidative stress in the intestine of pigs.

Therefore, the present study evaluated the effect of long-term supplementation with daidzein on the growth performance and antioxidant capacity of pigs. The antioxidant mechanism of daidzein was investigated with porcine intestinal epithelial cells (IPEC-J2), a non-transformed porcine intestinal epithelial cell line isolated from the jejunal epithelia of neonatal unsuckled piglets. We hypothesized that long-term supplementation with daidzein in corn–soybean meal diets was beneficial to the growth and antioxidant capacity of pigs, and daidzein exerted an antioxidant effect in IPEC-J2 cells. The results will have implications for the application of daidzein in pig production.

## 2 Materials and methods

### 2.1 Animal ethics

This study was approved by the Animal Care and Use Committee of the Feed Research Institute of the Chinese Academy of Agricultural Sciences.

### 2.2 Animals and experimental design

A total of 80 Large White × Landrace F1 crossbred piglets (40 barrows and 40 females) from a local commercial pig farm with similar initial BW (7.35 ± 0.14 kg) were weaned at 23 days of age and randomly assigned to 4 treatments with 5 pens per treatment and 4 piglets per pen according to BW and sex (half male and half female), for a 72-day trial. The dietary treatments included a corn–soybean meal basal diet supplemented with 0 (control group), 25, 50, or 100 mg/kg daidzein. The daidzein (purity ≥ 98%) used in this experiment was purchased from Guanghan Biochemical Products Co., Ltd. (Guanghan, China). The diets were formulated according to National Research Council (2012) nutrient requirements and the composition and nutrient levels in the basal diets are shown in Table 1. The barn was maintained at temperatures between 25 and 28 °C with a 12-h light/dark cycle. The pigs were allowed ad libitum access to water and feed throughout the experiment.

**Table 1:** Ingredients and nutrient composition of pigs' corn–soybean basal diets (%, as-fed basis).

Item	Pre-starter (day 0 to 14)	Starter (day 14 to 42)	Growing (day 42 to 72)
Ingredients			
Corn	22.00	41.12	64.25
Extruded corn	23.58	21.00	–
Soybean meal	15.00	21.00	20.00
Extruded soybean	14.50	4.00	4.00
Solvent rice bran meal	–	–	8.00
Fish meal	5.50	3.00	–
Whey	15.00	5.00	–
Soybean oil	0.50	0.70	–
Dicalcium phosphate	0.55	0.60	0.45
Limestone (CaCO_3_)	0.65	0.92	1.30
L-Lysine HCl	0.55	0.53	0.50
DL-Methionine	0.09	0.07	0.04
Threonine	0.08	0.06	0.08
Tryptophan	–	–	0.01
Salt	0.25	0.30	0.40
Choline chloride (60%)	0.08	0.08	–
Premix^1^	1.37	1.42	0.97
Zinc oxide	0.30	0.20	–
Analyzed nutrient content			
Dry matter	94.42	93.79	92.62
Crude protein	19.57	18.14	16.42
Calcium	0.91	0.83	0.75
Phosphorus	0.65	0.50	0.47
Calculated nutrient content			
ME, MJ/kg	14.35	13.93	13.67
Crude fat	5.20	3.84	3.43
Crude fiber	2.13	2.23	2.82
Lysine	1.31	1.15	0.98
Methionine	0.40	0.35	0.28
Threonine	0.73	0.65	0.59
Tryptophan	0.21	0.19	0.18

ME = metabolizable energy.

1 Premix supplied per kilogram of diet: vitamin A, 35.2 mg; vitamin D_3_, 7.68 mg; vitamin E, 128 mg; vitamin K_3_, 8.16 mg; vitamin B_1_, 4 mg; vitamin B_2_, 12 mg; vitamin B_6_, 8.32 mg; vitamin B_12_, 4.8 mg; niacin, 38.4 mg; calcium pantothenate, 25 mg; folic acid, 1.68 mg; biotin, 0.16 mg; iron (FeSO_4_·H_2_O), 171 mg; manganese (MnSO_4_·H_2_O), 42.31 mg; copper (CuSO_4_·5H_2_O), 125 mg; selenium (Na_2_SeO_3_), 0.19 mg; cobalt (CoCl_2_), 0.19 mg; iodine (Ca(IO_3_)_2_), 0.54 mg.

### 2.3 Sample collection

On days 14, 28, 42 and 72 of the trial, one male pig from each pen was selected randomly to collect blood samples via jugular veins. Then, blood samples were centrifuged at 3,000 × *g* for 10 min at 4 °C to obtain plasma; subsequently, the plasma was stored at −20 °C until analysis.

### 2.4 Growth performance measurement

Pigs were individually weighed on day 0 of the trial. However, BW by pen was measured on days 14, 28, 42 and 72 of the trial. Feed intake was recorded daily and the residual feed was measured when pigs were weighed. Growth performance was evaluated by calculating the average daily gain (ADG), average daily feed intake (ADFI) and feed conversion rate (FCR) for each pen.

### 2.5 Assay of plasma antioxidant indices

The activities of catalase (CAT), superoxide dismutase (SOD) and glutathione peroxidase (GSH-Px), and the content of malondialdehyde (MDA) in the plasma were measured using commercial assay kits (Nanjing Jiancheng Bioengineering Institute, Nanjing, China) according to the manufacturer's protocols. Briefly, the activities of CAT, SOD and GSH-Px, and the content of MDA were detected with the ammonium molybdate, non-enzymatic NBT test, 5,50-dithiobis-p-nitrobenzoic acid and 2-thiobarbituric acid, respectively. The absorbance changes were read at 405, 450, 412 and 532 nm for CAT, SOD, GSH-Px and MDA, respectively. Variation coefficients in intra-assay were 1.70%, 5.50%, 3.56% and 2.30% for CAT, SOD, GSH-Px and MDA, respectively. Variation coefficients in inter-assay were 1.70%, 3.32%, 6.80% and 5.34% for SOD, CAT, GSH-Px and MDA, respectively.

### 2.6 Cell culture

IPEC-J2 cells were obtained from Dr. Guoyao Wu's laboratory at Texas A&M University and cultured in Dulbecco's modified Eagle medium/F12 (DMEM/F12, Thermo Fisher Scientific, MA, USA) supplemented with 5% fetal bovine serum (FBS, Thermo Fisher Scientific, MA, USA), 0.1% ITS (5 μg/L insulin, 5 μg/L transferrin and 5 ng/L selenious acid, Corning Incorporated, NY, USA), 0.01% epidermal growth factor (5 μg/L, Corning Incorporated, NY, USA) and 1% pen-strep (Thermo Fisher Scientific, MA, USA) at 37 °C in a humidified atmosphere with 5% CO_2_.

### 2.7 Establishment of cell oxidative stress model

To select the optimal hydrogen peroxide (H_2_O_2_) concentration, IPEC-J2 cells were seeded at 1 × 10^5^ cells/mL (100 μL per well) in 96-well plates (Corning Incorporated, NY, USA) with 6 replications (wells) per treatment. After 48 h of incubation, oxidation was induced by exposing IPEC-J2 cells to 0, 0.2, 0.4, 0.6 and 0.8 mM H_2_O_2_ for a further 1 h. Subsequently, the supernatant was removed, the cells were washed twice with PBS (Thermo Fisher Scientific, MA, USA), and cell viability was determined using a cell counting kit (CCK-8, MedChemExpression, NJ, USA) according to the manufacturer's instructions. Briefly, 100 μL of FBS-free DMEM/F-12 (containing 10 μL of CCK-8 reagent) was added to each well. After 3 h of incubation at 37 °C, the absorbance was measured at 450 nm using an Epoch microplate spectrophotometer (BioTek Instruments Incorporated, VT, USA). Cell viability was calculated using the following equation: cell viability = (As − Ab)/(Ac − Ab) × 100%, where “As” represents the absorbance of the H_2_O_2_-treated group, “Ac” represents the absorbance of the H_2_O_2_ untreated group, and “Ab” represents the absorbance of the blank group, which contained culture medium and CCK-8 without cells and H_2_O_2_. The cell viability of the H_2_O_2_ untreated group was considered 100%. Due to 0.6 mM H_2_O_2_ leading to an approximately 31.4% loss in cell viability, this concentration was selected in our study to conduct the following experiments.

### 2.8 Selection of daidzein concentration

Daidzein was dissolved in dimethyl sulfoxide (DMSO, Sigma-Aldrich, St. Louis, MO, USA) at 10 mg/mL and diluted to the final concentration in a medium before use. To select the optimal daidzein concentration, IPEC-J2 cells were seeded at 1 × 10^5^ cells/mL (100 μL per well) in 96-well plates (Corning Incorporated, NY, USA) with 6 replications (wells) per treatment. After 24 h of incubation, daidzein at different concentrations (0, 20, 40, 60, 80, 100 μM) was added to the wells and incubated for another 24 h. In addition, the daidzein untreated group contained 0.2% DMSO. Then, 0.6 mM H_2_O_2_ was added to daidzein treated or untreated wells and incubated for 1 h. Cell viability was tested with the CCK-8 assay as described above. Because 40 μM daidzein led to higher cell viability, this concentration was selected in our study to carry out the following experiments.

### 2.9 Measurement of intracellular ROS

IPEC-J2 cells were seeded at 1 × 10^5^ cells/mL (100 μL per well) in 96-well plates (Corning Incorporated, NY, USA) with 6 replications (wells) per treatment, pretreated with or without 40 μM daidzein for 24 h, and then treated with or without 0.6 mM H_2_O_2_ for 1 h. At the end of the experiment, cells were incubated with DCFH-DA probes (Beyotime Biotechnology, Shanghai, China) for 30 min and then washed twice with PBS. The fluorescence was read at 488 nm for excitation and 525 nm for emission with a fluorescence microplate reader (Infinite M Plex, Tecan, Männedorf).

### 2.10 Measurements of SOD, CAT and GSH-Px activity and MDA content

IPEC-J2 cells were seeded at 1.5 × 10^5^ cells/mL (2 mL per well) in 6-well plates (Corning Incorporated, NY, USA) with 6 replications (wells) per treatment, pretreated with or without 40 μM daidzein for 24 h, and then treated with or without 0.6 mM H_2_O_2_ for 1 h. The supernatant was removed, and the cells were washed twice with ice-cold PBS and lysed by RIPA buffer (Thermo Fisher Scientific, MA, USA), which contained 1% protease inhibitors (Thermo Fisher Scientific, MA, USA) for 30 min at 4 °C. The supernatant was collected after centrifugation at 13,000 × *g* for 30 min at 4 °C. Commercial assay kits purchased from Nanjing Jiancheng Bioengineering Institute (Nanjing, China) were then used to detect SOD, CAT and GSH-Px activity and the content of MDA according to the manufacturer's instructions as described above.

### 2.11 RNA isolation, reverse transcription and quantitative real-time PCR (qRT-PCR)

IPEC-J2 cells were seeded at 1.5 × 10^5^ cells/mL (1 mL per well) in 12-well plates (Corning Incorporated, NY, USA) with 6 replications (wells) per treatment, pretreated with or without 40 μM daidzein for 24 h, and then treated with or without 0.6 mM H_2_O_2_ for 1 h. At the end of the experiment, cells were washed twice with ice-cold PBS; total RNA was extracted using TRIzol reagent (Thermo Fisher Scientific, MA, USA) in line with the manufacturer's protocol. The Epoch Microplate Spectrophotometer (BioTek Instruments Incorporated, VT, USA) was used to detect the content and quality of total RNA. cDNA was synthesized by reverse transcription using the TransScript First-Strand cDNA Synthesis Kit (TransGen Biotech, Beijing, China). SYBR Green reagent (Thermo Fisher Scientific, MA, USA) was used to perform the qRT-PCR on a real-time fluorescence quantitative system (Thermo Fisher Scientific, MA, USA). The gene expression of nuclear factor-erythroid 2-related factor 2 (*Nrf2*), superoxide dismutase 1 (*SOD1*), *CAT*, glutathione peroxidase 1 (*GPX1*), heme oxygenase-1 (*HO-1*), NAD(P)H: quinone oxidoreductase 1 (*NQO1*), zonula occludens-1 (*ZO-1*), occludin and claudin 1 was detected. Appendix Table 1 presents the detailed information of primers used for qRT-PCR. Glyceraldehyde-3-phosphate dehydrogenase (*GAPDH*) was selected as the housekeeping gene, and the expression of target genes relative to *GAPDH* was analyzed using the 2^−ΔΔCT^ method.

### 2.12 Immunofluorescence

IPEC-J2 cells were seeded at 1.5 × 10^5^ cells/mL (2 mL per well) in the confocal dish (Solarbio Life Sciences, Beijing, China) with 4 replications (wells) per treatment, pretreated with or without 40 μM daidzein for 24 h, and then treated with or without 0.6 mM H_2_O_2_ for 1 h. The cells were fixed with 4% paraformaldehyde for 20 min, permeabilized with 0.5% Triton X-100 for 20 min and blocked with 10% goat serum (Solarbio Life Sciences, Beijing, China) for 30 min. The cells were then incubated with Nrf2 antibody (Abcam, Cambridge, UK) overnight at 4 °C, followed by incubation with secondary antibody (FITC affinipure goat anti-rabbit IgG) for 30 min. DAPI staining was performed to define nuclear regions. The fluorescence images were captured by a laser-scanning confocal microscope (Carl Zeiss AG, Oberkochen, Germany). The fluorescence intensity was quantified using Image J software. Briefly, the merged image was opened in the Image J software, then the green channel image and blue channel image were obtained by splitting the channels. We selected the cells using the polygon drawing tool in the image of the green channel, added the selected cells into the region of interest (ROI) manager, followed by adjusting the threshold, then measured all the fluorescence values in the cell, the result values were the total fluorescence intensity of Nrf2 expressed in the cells. Subsequently, we adjusted the threshold in the image of the blue channel, then selected “Analyze particles” from the Analyze menu, the nuclei of the cells would be numbered, and added the number of the corresponding nucleus to the ROI manager, green fluorescence in the nuclear region was automatically selected and measured, the result values were the fluorescence intensity of Nrf2 expressed in the nuclei.

### 2.13 Western blotting

IPEC-J2 cells were seeded at 1.5 × 10^5^ cells/mL (2 mL per well) in 6-well plates (Corning Incorporated, NY, USA) with 4 replications (wells) per treatment, pretreated with or without 40 μM daidzein for 24 h, and then treated with or without 0.6 mM H_2_O_2_ for 1 h. At the end of the experiment, cells were washed twice with ice-cold PBS and lysed by RIPA buffer (Thermo Fisher Scientific, MA, USA), which contained 1% protease inhibitors (Thermo Fisher Scientific, MA, USA) for 30 min at 4 °C. The supernatant was collected after centrifugation at 13,000 × *g* for 30 min at 4 °C, then a BCA protein assay kit (Applygen, Beijing, China) was used to detect the protein content. Denaturation of proteins was accomplished by boiling 25 μg of protein and 4 × loading buffer (Bio-Rad Laboratories Incorporated, CA, USA) at 95 °C for 10 min. 12% SDS-PAGE was used to separate the denatured protein samples, then transferred to PVDF membranes (Bio-Rad Laboratories Incorporated, CA, USA) for 2 h at 200 mA. After blocking for 3 h at room temperature with 5% skim milk in Tris-buffered saline with Tween 20 (TBST), the membranes were incubated with primary antibodies (ZO-1, occludin and GAPDH) at 4 °C overnight. The membranes were washed with TBST 3 times and then treated with secondary antibodies for 1 h at room temperature. The membranes were washed again and visualized using an ECL agent (Bio-Rad Laboratories Incorporated, CA, USA). The ChemiDoc MP Imaging System (Bio-Rad Laboratories Incorporated, CA, USA) was used to measure the images. GAPDH was used as an internal reference. Antibodies used in western blotting are shown in Appendix Table 2.

### 2.14 Statistical analysis

Data related to growth performance were analyzed by ANOVA using a completely randomized block design using the general linear model (GLM) procedure of SPSS 20.0. The remaining data were analyzed using the one-way ANOVA procedure of SPSS 20.0. The assumptions of normality of error were confirmed post-hoc. The pen represents the experimental unit for growth performance. Tukey's multiple comparison test was used to determine treatment differences. Significant differences among the treatments were determined at *P* < 0.05, whereas a treatment effect trend was noted for 0.05 < *P* < 0.10.

## 3 Results

### 3.1 Growth performance

As shown in Table 2, there was no significant effect on BW on days 0, 14, 28 and 42, or on ADG during days 0 to 14, 14 to 28, 28 to 42 and 42 to 72; ADFI and FCR were observed (*P* > 0.05). Compared with the control diet, supplementation with daidzein at 50 mg/kg increased BW on day 72 and ADG during days 0 to 72 (*P* < 0.05). In addition, compared with pigs fed dietary daidzein at 25 mg/kg, that fed dietary daidzein at 50 mg/kg tended to increase BW on day 72 (*P* = 0.088) and ADG during days 0 to 72 (*P* = 0.085), while there was no significant difference on BW on day 72 and ADG during days 0 to 72 between the group supplemented with 100 mg/kg daidzein and the other three groups (*P* > 0.05). Moreover, both linear and quadratic effects were not observed (*P* > 0.05).

**Table 2:** Effect of daidzein on growth performance of pigs.^1^

Item	Daidzein, mg/kg				SEM	*P*-value		
	0	25	50	100		ANOVA	Linear	Quadratic
BW, kg								
Day 0	7.41	7.34	7.33	7.33	0.555	0.366	0.923	0.952
Day 14	10.26	10.12	10.33	10.07	0.696	0.778	0.910	0.934
Day 28	16.22	16.06	16.96	16.36	1.098	0.493	0.790	0.845
Day 42	22.67	23.12	24.70	23.90	1.536	0.320	0.465	0.697
Day 72	41.78^b^	42.53^ab, y^	45.98^a, x^	43.45^ab^	1.920	0.037	0.349	0.414
ADG^2^, g								
Day 0 to 14	204	199	214	196	14.6	0.797	0.904	0.656
Day 14 to 28	426	424	474	450	31.7	0.311	0.425	0.734
Day 28 to 42	461	504	553	538	42.9	0.410	0.185	0.531
Day 42 to 72	637	647	709	652	19.8	0.109	0.319	0.159
Day 0 to 72	477^b^	489^ab, y^	537^a, x^	502^ab^	20.0	0.033	0.211	0.277
ADFI^3^, g								
Day 0 to 14	328	321	334	308	23.5	0.756	0.664	0.693
Day 14 to 28	753	745	769	730	50.8	0.753	0.847	0.760
Day 28 to 42	920	960	1,026	983	90.6	0.716	0.558	0.673
Day 42 to 72	1,391	1,423	1,556	1,436	61.7	0.102	0.368	0.253
Day 0 to 72	968	987	1,062	991	52.1	0.152	0.557	0.414
FCR^4^								
Day 0 to 14	1.62	1.61	1.56	1.58	0.040	0.712	0.396	0.815
Day 14 to 28	1.81	1.76	1.63	1.62	0.066	0.264	0.056	0.836
Day 28 to 42	2.06	1.88	1.88	1.83	0.114	0.667	0.233	0.585
Day 42 to 72	2.18	2.20	2.19	2.21	0.063	0.995	0.847	0.957
Day 0 to 72	2.03	2.01	1.98	1.98	0.036	0.649	0.296	0.877

SEM = standard error of the mean; BW = body weight.

^a, b^Values within a row without common letters differ significantly (*P* < 0.05).

^x, y^Values listed in the same row with different superscripts are tended to be different (0.05 < *P* < 0.10).

1 Five replicates per treatment (*n* = 5); the total number of animals is 80.

2 Average daily gain (ADG) = (body weight gain of the pen/piglets' number)/days.

3 Average daily feed intake (ADFI) = (feed intake of the pen/piglets' number)/days.

4 Feed conversion rate (FCR) = feed intake of the pen/body weight gain of the pen.

### 3.2 Antioxidant capacity

According to Table 3, there was no significant effect on plasma SOD activity on days 14, 28 and 72 or MDA content on days 28, 42 and 72, and CAT and GSH-Px activity (*P* > 0.05). Compared with the control group, dietary supplementation with 50 mg/kg daidzein enhanced plasma SOD activity on day 42 (*P* < 0.05) and tended to decrease plasma MDA content on day 14 (*P* = 0.062). In addition, compared with pigs fed dietary daidzein at 25 mg/kg, that fed dietary daidzein at 50 mg/kg tended to decrease plasma MDA content on day 14 (*P* = 0.062). However, there was no significant difference in plasma SOD activity on day 42 between the group supplemented with 100 mg/kg daidzein and the other three groups (*P* > 0.05).

**Table 3:** Effect of daidzein on plasma antioxidant capacity of pigs.^1^

Item	Daidzein, mg/kg				SEM	*P*-value		
	0	25	50	100		ANOVA	Linear	Quadratic
Day 14								
CAT, U/mL	12.56	12.76	12.99	12.93	0.348	0.833	0.419	0.719
SOD, U/mL	19.61	19.33	19.21	19.19	0.748	0.979	0.690	0.868
MDA, nmol/mL	2.52^x^	2.54^x^	2.31^y^	2.50^xy^	0.055	0.040	0.250	0.109
GSH-Px, U/mL	493	479	499	490	21.8	0.930	0.918	0.924
Day 28								
CAT, U/mL	12.51	12.82	12.99	12.71	0.447	0.892	0.711	0.514
SOD, U/mL	22.06	21.87	22.04	21.29	0.527	0.742	0.404	0.621
MDA, nmol/mL	2.58	2.47	2.56	2.23	0.143	0.341	0.180	0.430
GSH-Px, U/mL	499	492	517	509	15.7	0.741	0.474	0.985
Day 42								
CAT, U/mL	12.31	12.43	13.17	12.67	0.544	0.702	0.469	0.586
SOD, U/mL	18.19^b^	18.58^ab^	20.54^a^	19.01^ab^	0.544	0.042	0.094	0.098
MDA, nmol/mL	2.07	2.05	1.98	2.02	0.155	0.986	0.765	0.866
GSH-Px, U/mL	510	588	566	559	29.6	0.332	0.368	0.169
Day 72								
CAT, U/mL	9.52	10.36	10.72	10.78	0.679	0.591	0.214	0.599
SOD, U/mL	14.42	15.64	14.53	15.05	0.526	0.403	0.755	0.536
MDA, nmol/mL	3.23	3.29	3.21	3.42	0.208	0.902	0.630	0.719
GSH-Px, U/mL	412	430	421	415	19.4	0.924	0.977	0.552

SEM = standard error of the mean; CAT = catalase; SOD = superoxide dismutase; MDA = malondialdehyde; GSH-Px = glutathione peroxidase.

^a, b^Values within a row without common letters differ significantly (*P* < 0.05).

^x, y^Values listed in the same row with different superscripts are tended to be different (0.05 < *P* < 0.10).

1 Five replicates per treatment (*n* = 5).

### 3.3 The concentration of H_2_O_2_ in IPEC-J2 cells

**Fig. 1:**
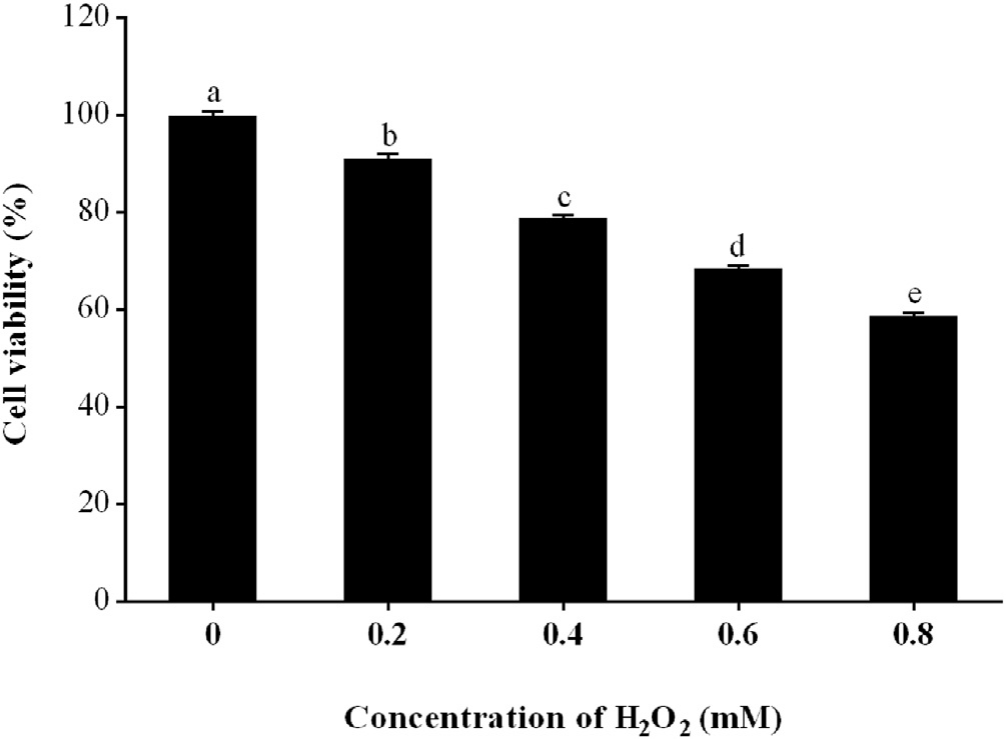
The viability of IPEC-J2. IPEC-J2 cells were seeded in 96-well plates and treated with 0 to 0.8 mM H_2_O_2_ for 1 h after 48 h of incubation. The results are presented as the mean ± SE, *n* = 6. The value is expressed as a percentage of the H_2_O_2_ untreated group. Values without common letters (a, b, c, d, e) differ significantly (*P* < 0.05). IPEC-J2 = porcine intestinal epithelial cells; H_2_O_2_ = hydrogen peroxide.

Concentrations of 0.2, 0.4, 0.6 and 0.8 mM H_2_O_2_ significantly inhibited cell viability compared to the untreated group (Fig. 1; *P* < 0.05), reducing cell viability from 100% to 91.2%, 78.9%, 68.6% and 58.8%, respectively. Since 0.6 mM H_2_O_2_ led to an approximately 31.4% loss in cell viability, this concentration was selected in our study to conduct the following experiments.

### 3.4 The concentration of daidzein in IPEC-J2 cells

**Fig. 2:**
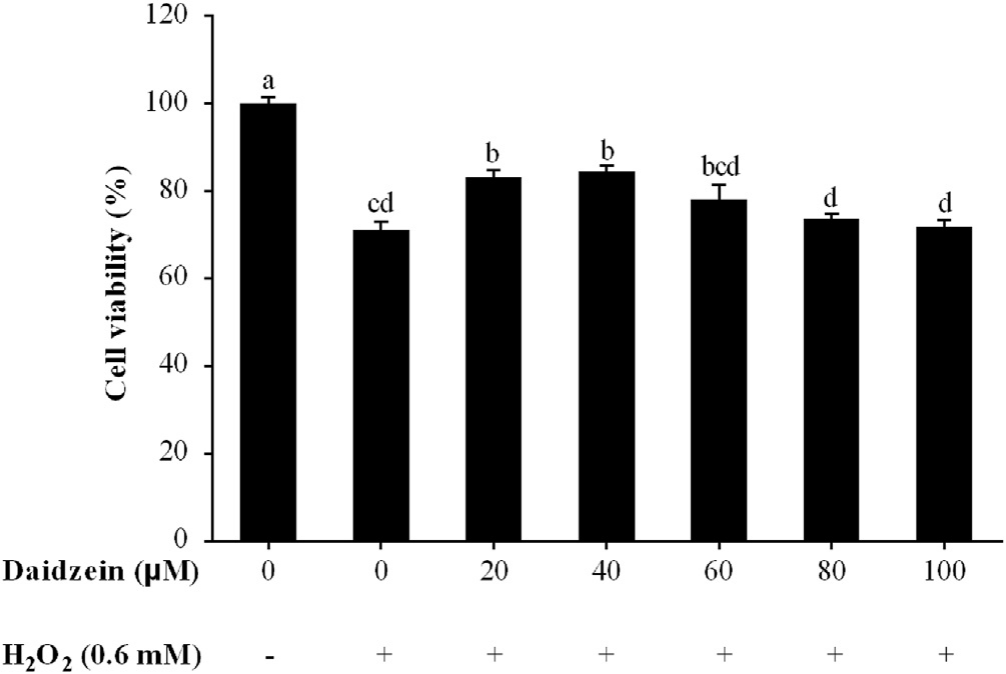
Effect of daidzein on the viability of IPEC-J2 cells. IPEC-J2 cells were seeded in 96-well plates, pretreated with different daidzein concentrations (0 to 100 μM) for 24 h, and then treated with or without 0.6 mM H_2_O_2_ for 1 h. The results are presented as the mean ± SE, *n* = 6. The value is expressed as a percentage of the group not treated with H_2_O_2_ or daidzein. Values without common letters (a, b, c, d) differ significantly (*P* < 0.05). IPEC-J2 = porcine intestinal epithelial cells; H_2_O_2_ = hydrogen peroxide.

Pretreatment with 20 and 40 μM daidzein restored cell viability from 71.2% to 83.1% and 84.5%, respectively (Fig. 2, *P* < 0.05). However, compared with the H_2_O_2_-treated group, pretreatment with 60, 80 and 100 μM daidzein prior to H_2_O_2_ exposure did not improve cell viability (*P* > 0.05). Because 40 μM daidzein led to higher cell viability, the concentration of 40 μM was selected in our study to carry out the following experiments.

### 3.5 Intracellular ROS

**Fig. 3:**
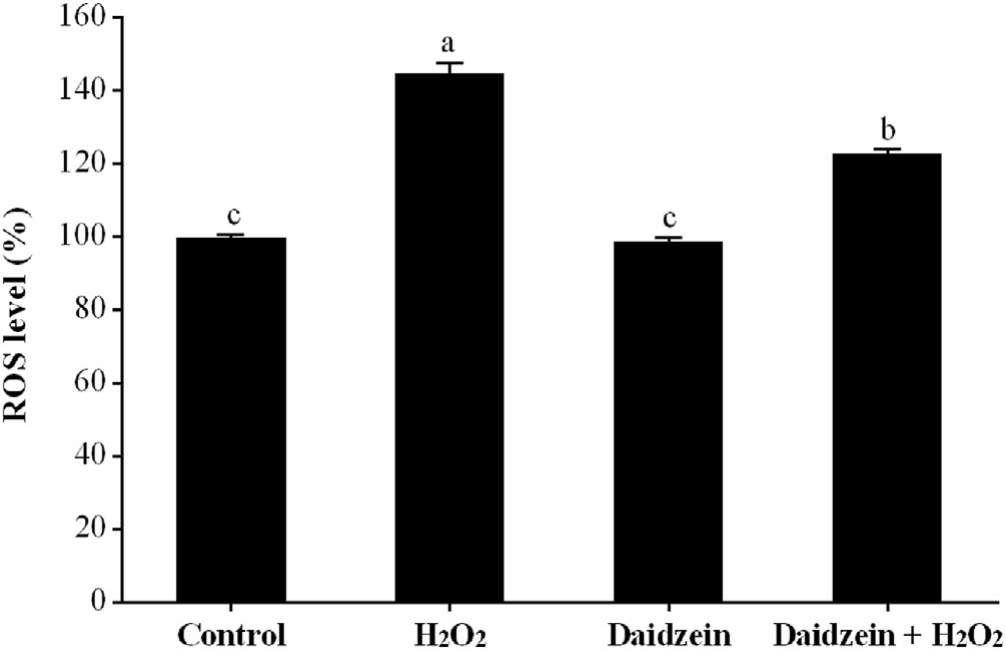
Effect of daidzein on ROS levels in IPEC-J2 cells. IPEC-J2 cells were seeded in 96-well plates, pretreated with or without 40 μM daidzein for 24 h, treated with or without 0.6 mM H_2_O_2_ for 1 h, and subsequently incubated with DCFH-DA probes for 30 min. The results are presented as the mean ± SE, *n* = 6. Values without common letters (a, b, c) differ significantly (*P* < 0.05). IPEC-J2 = porcine intestinal epithelial cells; H_2_O_2_ = hydrogen peroxide; ROS = reactive oxygen species.

As shown in Fig. 3, the H_2_O_2_-treated group had significantly increased intracellular ROS levels compared to the control group (*P* < 0.05). Pretreatment with 40 μM daidzein prior to H_2_O_2_ exposure significantly decreased intracellular ROS levels compared to the H_2_O_2_-treated group (*P* < 0.05). However, daidzein treatment alone did not affect intracellular ROS levels compared to the control group (*P* > 0.05).

### 3.6 SOD, CAT and GSH-Px activity and MDA content in H_2_O_2_-treated IPEC-J2 cells

**Fig. 4:**
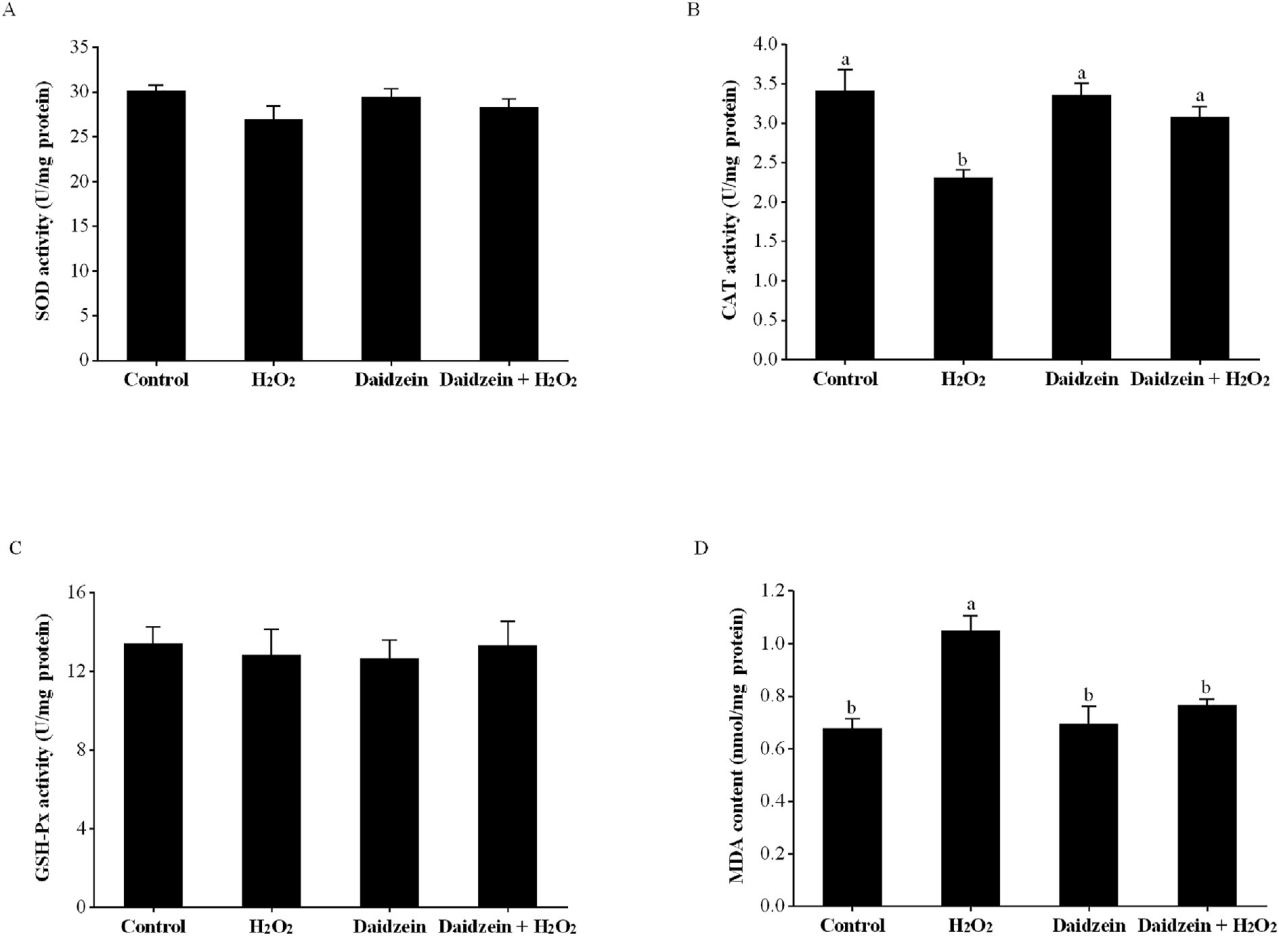
Effects of daidzein on antioxidant enzyme activity and MDA content in IPEC-J2 cells. (A) SOD activity; (B) CAT activity; (C) GSH-Px activity; (D) MDA content. IPEC-J2 cells were seeded in 6-well plates, pretreated with or without 40 μM daidzein for 24 h, and then treated with or without 0.6 mM H_2_O_2_ for 1 h. The results are presented as the mean ± SE, *n* = 6. Values without common letters (a, b) differ significantly (*P* < 0.05). IPEC-J2 = porcine intestinal epithelial cells; H_2_O_2_ = hydrogen peroxide; SOD = superoxide dismutase; CAT = catalase; GSH-Px = glutathione peroxidase; MDA = malondialdehyde.

As displayed in Fig. 4, the H_2_O_2_-treated group had significantly decreased CAT activity and increased MDA content compared to the control group (*P* < 0.05). Pretreatment with 40 μM daidzein prior to H_2_O_2_ exposure significantly increased CAT activity and decreased MDA content compared to the H_2_O_2_-treated group (*P* < 0.05), whereas daidzein treatment alone did not affect CAT activity and MDA content compared to the control group (*P* > 0.05). In addition, no significant difference in SOD and GSH-Px activity among the groups was observed (*P* > 0.05).

### 3.7 Gene expression of Nrf2 signaling pathway in H_2_O_2_-treated IPEC-J2 cells

**Fig. 5:**
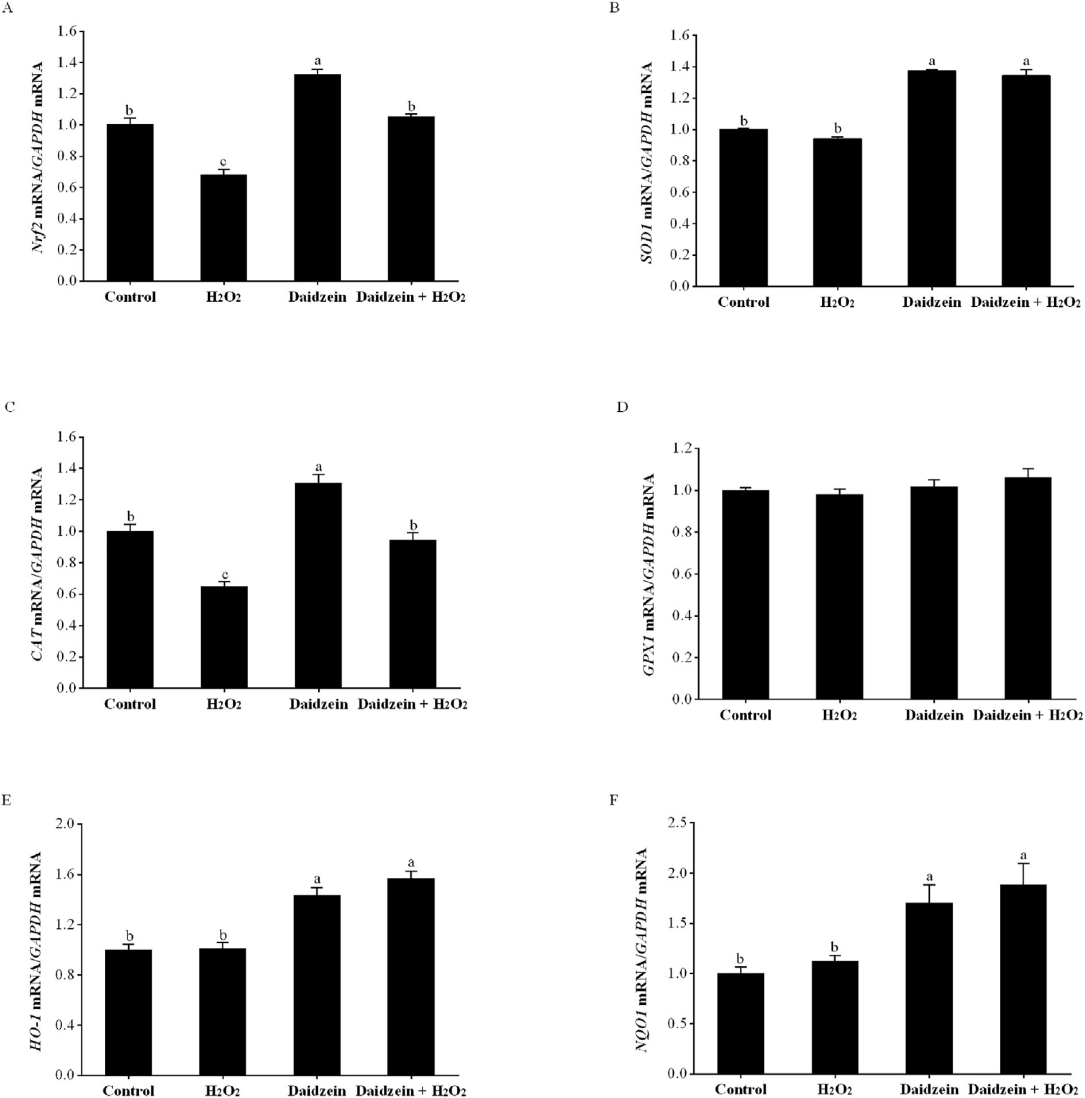
Effect of daidzein on the gene expression of Nrf2 signaling pathway in IPEC-J2 cells. (A) Gene expression of *Nrf2*; (B) Gene expression of *SOD1*; (C) Gene expression of *CAT*; (D) Gene expression of *GPX1*; (E) Gene expression of *HO-1*; (F) Gene expression of *NQO1*. IPEC-J2 cells were seeded in 12-well plates, pretreated with or without 40 μM daidzein for 24 h, and then treated with or without 0.6 mM H_2_O_2_ for 1 h. The results are presented as the mean ± SE, *n* = 6. Values without common letters (a, b, c) differ significantly (*P* < 0.05). IPEC-J2 = porcine intestinal epithelial cells; H_2_O_2_ = hydrogen peroxide; *GAPDH* = glyceraldehyde-3-phosphate dehydrogenase; *Nrf2* = nuclear factor-erythroid 2-related factor 2; *SOD1* = superoxide dismutase 1; *CAT* = catalase; *GPX1* = glutathione peroxidase 1; *HO-1* = heme oxygenase-1; *NQO1* = NAD(P)H: quinone oxidoreductase 1.

According to Fig. 5, the H_2_O_2_-treated group had significantly decreased gene expression of *Nrf2* and *CAT* compared to the control group (*P* < 0.05). However, pretreatment with 40 μM daidzein prior to H_2_O_2_ exposure significantly increased the gene expression of *Nrf2*, *SOD1*, *CAT, HO-1* and *NQO1* compared to that of the H_2_O_2_-treated group (*P* < 0.05). In addition, daidzein treatment alone significantly enhanced the gene expression of *Nrf2*, *SOD1*, *CAT*, *HO-1* and *NQO1* compared to the control group (*P* < 0.05). No significant difference in the gene expression of *GPX1* among the groups was observed (*P* > 0.05).

### 3.8 Gene expression of tight junctions in H_2_O_2_-treated IPEC-J2 cells

**Fig. 6:**
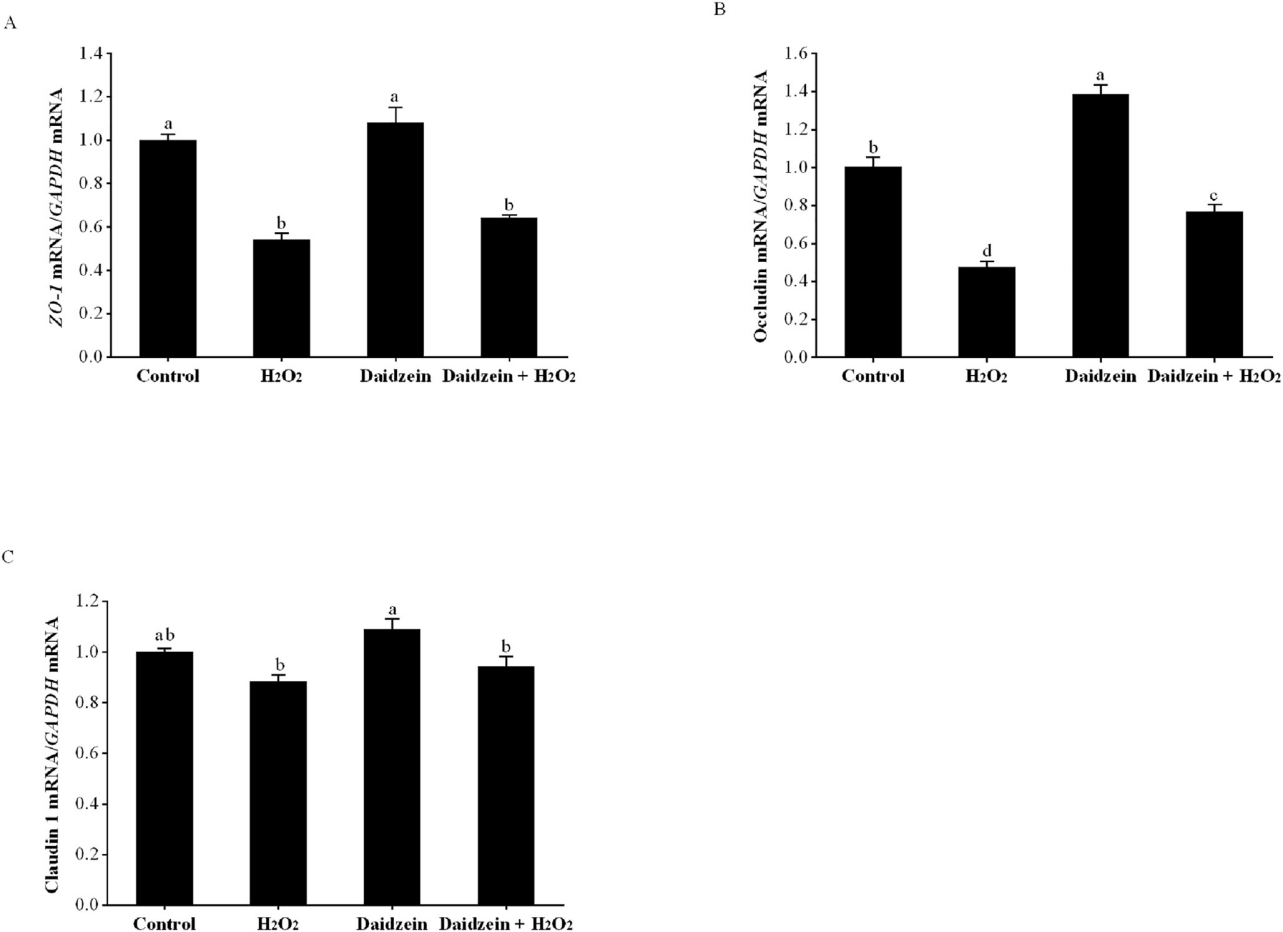
Effect of daidzein on the gene expression of tight junctions in H_2_O_2_-treated IPEC-J2 cells. (A) Gene expression of *ZO-1*; (B) Gene expression of occludin; (C) Gene expression of claudin 1. IPEC-J2 cells were seeded in 12-well plates, pretreated with or without 40 μM daidzein for 24 h, and then treated with or without 0.6 mM H_2_O_2_ for 1 h. The results are presented as the mean ± SE, *n* = 6. Values without common letters (a, b, c, d) differ significantly (*P* < 0.05). IPEC-J2 = porcine intestinal epithelial cells; H_2_O_2_ = hydrogen peroxide; *GAPDH* = glyceraldehyde-3-phosphate dehydrogenase; *ZO-1* = zonula occludens-1.

As presented in Fig. 6, compared to the control group, the H_2_O_2_-treated group had significantly decreased gene expression of *ZO-1* and occludin (*P* < 0.05), but no difference in the gene expression of claudin 1 (*P* > 0.05). Pretreatment with 40 μM daidzein prior to H_2_O_2_ exposure significantly increased the gene expression of occludin (*P* < 0.05), whereas it did not improve the gene expression of *ZO-1* and claudin 1 compared to that in the H_2_O_2_-treated group (*P* > 0.05). In addition, daidzein treatment alone significantly enhanced the gene expression of occludin compared to the control group (*P* < 0.05), while it did not affect the gene expression of *ZO-1* and claudin 1 (*P* > 0.05).

### 3.9 Protein abundance of Nrf2 in H_2_O_2_-treated IPEC-J2 cells

**Fig. 7:**
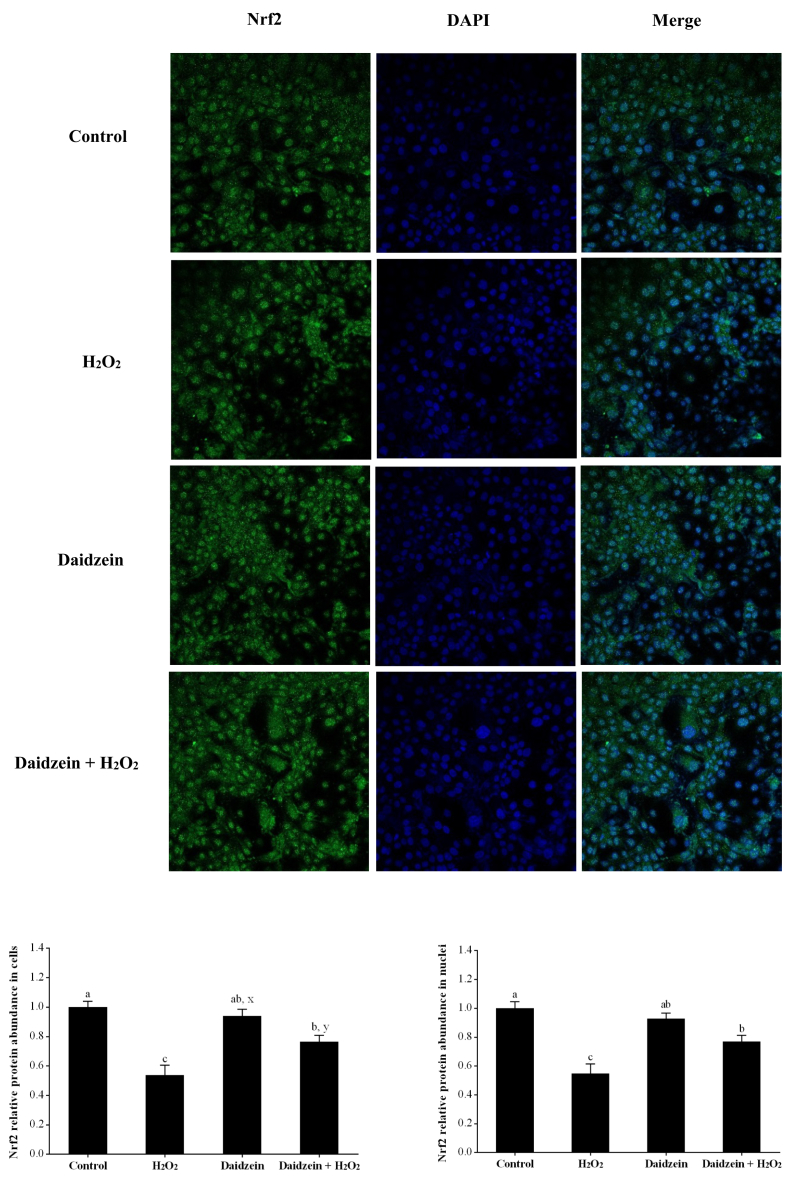
Effect of daidzein on the relative protein abundance of Nrf2 in IPEC-J2 cells and nuclei. IPEC-J2 cells were seeded in the confocal dish, pretreated with or without 40 μM daidzein for 24 h, and then treated with or without 0.6 mM H_2_O_2_ for 1 h. The results are presented as the mean ± SE, *n* = 4. Values without common letters (a, b, c) differ significantly (*P* < 0.05), and different superscripts (x, y) are tended to be different (0.05 < *P* < 0.10). IPEC-J2 = porcine intestinal epithelial cells; H_2_O_2_ = hydrogen peroxide; Nrf2 = nuclear factor-erythroid 2-related factor 2.

As demonstrated in Fig. 7, the H_2_O_2_-treated group had significantly decreased protein abundance of Nrf2 in cells and nuclei compared to the control group (*P* < 0.05). However, pretreatment with 40 μM daidzein prior to H_2_O_2_ exposure significantly increased the protein abundance of Nrf2 in cells and nuclei compared to that in the H_2_O_2_-treated group (*P* < 0.05). In addition, compared to the control group, daidzein treatment alone did not affect the protein abundance of Nrf2 (*P* > 0.05).

### 3.10 Protein abundance of tight junctions in H_2_O_2_-treated IPEC-J2 cells

**Fig. 8:**
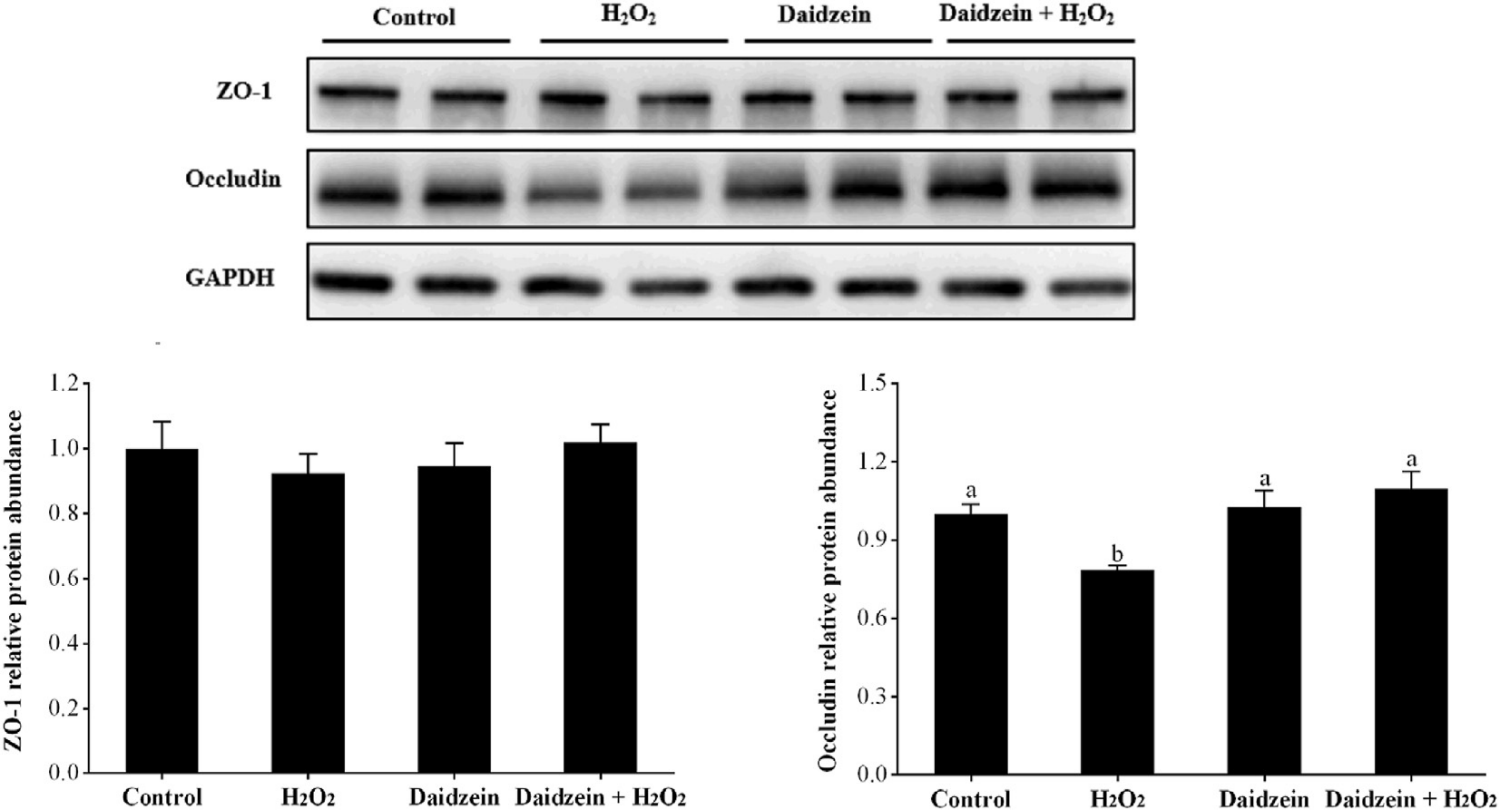
Effect of daidzein on the relative protein abundance of tight junctions in H_2_O_2_-treated IPEC-J2 cells. IPEC-J2 cells were seeded in 6-well plates, pretreated with or without 40 μM daidzein for 24 h, and then treated with or without 0.6 mM H_2_O_2_ for 1 h. The results are presented as the mean ± SE, *n* = 4. The value is expressed as a percentage of the control group. Values without common letters (a, b) differ significantly (*P* < 0.05). IPEC-J2 = porcine intestinal epithelial cells; H_2_O_2_ = hydrogen peroxide; GAPDH = glyceraldehyde-3-phosphate dehydrogenase; ZO-1 = zonula occludens-1.

As illustrated in Fig. 8, the H_2_O_2_-treated group exhibited a significantly lower protein abundance of occludin than the control group (*P* < 0.05). However, pretreatment with 40 μM daidzein prior to H_2_O_2_ exposure significantly increased the protein abundance of occludin compared to that in the H_2_O_2_-treated group (*P* < 0.05). However, no significant difference in the protein abundance of ZO-1 among the groups was observed (*P* > 0.05).

## 4 Discussion

In the present study, dietary supplementation with 50 mg/kg of daidzein significantly improved the growth performance of pigs during days 0 to 72 of the trial, indicating that long-term addition of daidzein to a corn–soybean diet benefits pig growth. This result corroborates our previous study in which soybean isoflavones in soybean meal were proven to play important roles in enhancing growth performance in pigs (Li et al., 2020). Our results also agree with other studies on the beneficial effects of daidzein on growth performance. Sun et al. (2020) found that 37.5 and 62.5 mg/kg of daidzein significantly enhanced the ADG of growing-finishing pigs. Greiner et al. (2001) observed that 200 or 400 mg/kg of daidzein could improve body growth in porcine reproductive and respiratory syndrome virus-infected pigs. However, other studies showed no growth-promoting effect with daidzein addition. Adding different concentrations of daidzein to the diet without any soy source did not affect pig growth (Xiao et al., 2015). Growing-finishing pigs' growth was not significantly affected when supplementing 2 or 5 times isoflavone as high as the corn–soybean meal diet (Payne et al., 2001). In addition, virus-challenged pigs' growth was not affected when increasing the level of soybean meal in the diet (Rochell et al., 2015). The differences in our results may be due to the exposure time or level of isoflavone in the diet, the pig's feeding phase, or the specific composition of the diet. The corn–soybean diet was used in our experiment, whereas the diet without any soy source was used in the study of Xiao et al. (2015). The pigs started feeding with daidzein from weaning in our trial, while the pig's feeding phase related to isoflavone was initial BW (26 kg) to final BW (113 kg) or initial BW (31 kg) to final BW (116 kg) in the study of Payne et al. (2001). In addition, the exposure time to daidzein is longer in our study (72 days) than that in the research of Rochell et al. (2015) (28 days).

Daidzein is a polyphenol compound, and the hydrogen atoms in the phenolic hydroxyl group can react with free radicals, scavenging free radicals (Liang et al., 2008; Wijeratne and Cuppett, 2007). Several studies reported the antioxidant capacity of daidzein. The activities of CAT, SOD and GSH-Px, and the content of MDA reflect the antioxidant and lipid peroxidation status of animal tissues and cultured cells (Efe et al., 1999). Sun et al. (2020) indicated that dietary daidzein significantly elevated the activities of CAT and T-SOD and reduced the MDA level in the serum of growing-finishing pigs. Zheng et al. (2014) demonstrated that liver Mn-SOD activity of barrows was significantly enhanced after adding 50 mg/kg of daidzein to an isoflavone-free basal diet. Xiao et al. (2015) found that dietary daidzein (200 mg/kg) significantly improved the serum SOD activity and decreased the serum MDA level of pigs. Similar results were reported in the studies of Liu et al. (2013), Zhao et al. (2017) and Zhang et al. (2018) using late lactation cows, bull calves and rats, respectively. These results were consistent with our study in which pigs fed a diet supplemented with 50 mg/kg daidzein had increased SOD activity and decreased MDA content in the plasma. However, the antioxidative mechanism of daidzein remains unclear.

To explore the mechanism underlying antioxidation by daidzein, we employed an in vitro model with the IPEC-J2 cell line, a non-transformed porcine intestinal epithelial cell line, with H_2_O_2_ stimulation mimicking oxidative stress (Qin et al., 2019; Zhu et al., 2019).

Under normal physiological conditions, the antioxidant system of the body maintains a balance between the generation and elimination of ROS. However, ROS levels dramatically increase under oxidative stress (Liu et al., 2010) and it has been reported that ROS production is related to cell damage and death (Newsholme et al., 2007); thus, ROS production is a vital indicator of oxidative stress (Datta et al., 2017). In addition, as described above, the activities of CAT, SOD and GSH-Px, and the content of MDA reflect the antioxidant status of cultured cells (Efe et al., 1999). In the present study, exposure of IPEC-J2 cells to H_2_O_2_ significantly enhanced ROS levels. Furthermore, CAT activity significantly decreased, while MDA content significantly increased after H_2_O_2_ treatment. These results demonstrate that the oxidative stress model was successfully established. In addition, daidzein pretreatment followed by H_2_O_2_ exposure remarkably decreased ROS levels, increased CAT activity and decreased MDA content. This observation was in accordance with previous studies. Rotenone significantly induced excess ROS in HUVECs, and pretreatment with kudzu root extract (containing daidzein) attenuated the ROS levels in HUVECs exposed to rotenone (Gao et al., 2016). ROS generation induced by the high glucose treatment was significantly reduced in the presence of daidzein in HUVECs (Park et al., 2016). Pretreatment with daidzein reduced ROS production and enhanced SOD activity in hepatocytes induced by lipopolysaccharide (Yu et al., 2020). These findings indicated that daidzein could act as a potent antioxidant against oxidative stress.

The Nrf2 signaling pathway plays an important role in preventing oxidative stress in cells (Kubben et al., 2016; Xu et al., 2019). Under normal physiological conditions, *Nrf2* is mainly located in the cytoplasm and binds to Kelch-like ECH-associated protein 1 (*Keap1*). Due to proteasomal degradation mediated by *Keap1*, *Nrf2* is inactive. Under oxidative stress, the cysteine residues of *Keap1* can be modified and its conformational changes result in a decrease in its binding affinity to *Nrf2*. Subsequently, activated *Nrf2* translates from the cytoplasm to the nucleus, specifically binds to the antioxidant response element, promotes the expression of downstream antioxidant enzymes (*SOD*, *CAT* and *GPX1*) and detoxifying enzymes (*HO-1* and *NQO1*) genes, and enhances the antioxidant capacity of the body to resist the injury caused by oxidative stress (Tang et al., 2014). In the present study, we observed that Nrf2 protein was mainly expressed in the nucleus of the cells; daidzein increased the total and nuclear Nrf2 protein levels when IPEC-J2 cells were stimulated by H_2_O_2_ and led to the activation of antioxidant enzymes and detoxifying enzymes. This demonstrated that daidzein enhanced Nrf2 protein expression and upregulated the expression of antioxidant enzymes and detoxifying enzymes to reduce H_2_O_2_-induced oxidative injury in IPEC-J2 cells. Similar results were observed in the study that daidzein exerted antioxidant effects against lipopolysaccharide-induced hepatocyte injury by upregulating *Nrf2* expression (Yu et al., 2020).

As we mentioned above, daidzein effectively reduced the ROS level induced by H_2_O_2_, mitigated the increase of MDA and recovered the downregulation of CAT activity and Nrf2 protein caused by H_2_O_2_ in IPEC-J2 cells. However, dietary supplementation with daidzein only had minor effects on plasma antioxidant indices. These results indicated that daidzein might have more of an effect on the antioxidant capacity of local intestinal cells than systemic antioxidant capacity. Future study is needed to determine if dietary daidzein affects the antioxidant indices of intestinal mucosa in piglets.

As a key component of the intestinal mucosal epithelial barrier, tight junctions are closely related to intestinal permeability (Groschwitz and Hogan, 2009; Banan et al., 2005). Increased intestinal permeability provides inflammatory and infectious agents opportunities to enter systemic circulation and cause tissue damage (Omonijo et al., 2019). As crucial proteins in tight junctions, ZO-1, occludin and claudin 1 play an important role in intestinal health (He et al., 2019). Hu et al. (2013) and Zhang and Guo (2009) reported that weaned piglets' intestinal permeability was reduced with the expression of *ZO-1* and occludin increasing. In the present study, the exposure of IPEC-J2 cells to H_2_O_2_ significantly decreased the gene expression of occludin and *ZO-1* and the protein abundance of occludin, while daidzein pretreatment followed by H_2_O_2_ exposure significantly increased the gene expression and protein abundance of occludin. Similarly, in the study of Kang et al. (2019), the *Pueraria lobata* extract (containing daidzein) attenuated the decrease in the expression of *ZO-1* in H_2_O_2_-treated human retinal pigment epithelial cells. These results indicated that daidzein elicited a protective effect on intestinal barrier function.

## 5 Conclusions

In conclusion, adding 50 mg/kg daidzein to a corn–soybean basal diet improved BW on day 72 and ADG during days 0 to 72 in pigs, and was beneficial to the antioxidant capacity of pigs. Daidzein had a protective effect on IPEC-J2 cells against H_2_O_2_-induced oxidative stress. The mechanism by which daidzein enhances antioxidant capacity may be related to the activation of the Nrf2 signaling pathway in IPEC-J2 cells.

## Author contributions

**Yanpin Li**: acquisition, analysis of data, drafting manuscript; **Xianren Jiang**: data analysis, critically revising the manuscript; **Long Cai**: acquisition of data; **Yanli Zhang**: acquisition of data; **Hongbiao Ding**: critically revising the manuscript; **Jingdong Yin**: critically revising the manuscript; **Xilong Li**: design of the experiment, data analysis, and critically revising the manuscript.

## Declaration of competing interest

We declare that we have no financial and personal relationships with other people or organizations that can inappropriately influence our work, and there is no professional or other personal interest of any nature or kind in any product, service and/or company that could be construed as influencing the content of this paper.
