# Supplementary material for: Effects of daidzein on antioxidant capacity in weaned pigs and IPEC-J2 cells
Source: Anim Nutr. 2022 Jul 14;11:48–59. doi: 10.1016/j.aninu.2022.06.014 (PMC9428850; doi:10.1016/j.aninu.2022.06.014)
Supplement: Multimedia component 1 [file mmc1.docx]

**Appendix Table 1** Primer sequences used for quantitative real-time PCR.

| Gene^1^ | Forward (5´- 3´) | Reverse (5´- 3´) | Product length, bp | Accession no. |
| --- | --- | --- | --- | --- |
| *GAPDH* | GCTTGTCATCAATGGAAAGG | CATACGTAGCACCAGCATCA | 86 | NM_001206359.1 |
| *SOD1* | GAAGACAGTGTTAGTAACGG | CAGCCTTGTGTATTATCTCC | 93 | NM_001190422.1 |
| *CAT* | CCTGCAACGTTCTGTAAGGC | GCTTCATCTGGTCACTGGCT | 72 | NM_214301.2 |
| *GPX1* | TCTCCAGTGTGTCGCAATGA | TCGATGGTCAGAAAGCGACG | 104 | NM_214201.1 |
| *Nrf2* | GACCTTGGAGTAAGTCGAGA | GGAGTTGTTCTTGTCTTTCC | 103 | XM_005671981.3 |
| *HO-1* | GAGAAGGCTTTAAGCTGGTG | GTTGTGCTCAATCTCCTCCT | 74 | NM_001004027.1 |
| *NQO1* | GGACATCACAGGTAAACTGA | TATAAGCCAGAGCAGTCTCG | 68 | NM_001159613.1 |
| *Occludin* | TCAGGTGCACCCTCCAGATT | TGGACTTTCAAGAGGCCTGG | 112 | NM_001163647.2 |
| *ZO-1* | CGATCACTCCAGCATACAAT | CACTTGGCAGAAGATTGTGA | 111 | CV870309 |
| *Claudin 1* | CCTCAATACAGGAGGGAAGC | CTCTCCCCACATTCGAGATGATT | 76 | NM_001244539.1 |

^1^*GAPDH* = glyceraldehyde-3-phosphate dehydrogenase; *SOD1* = superoxide dismutase 1; *CAT* = catalase; *GPX1* = glutathione peroxidase 1; *Nrf2* = nuclear factor-erythroid2-related factor 2; *HO-1* = heme oxygenase-1; *NQO1* = NAD(P)H: quinone oxidoreductase 1; *ZO-1* = zonula occludens-1.

**Appendix Table 2** Antibodies used in western blotting.

| Antibody^1^ | Source | Dilution | Company | Cat# |
| --- | --- | --- | --- | --- |
| ZO-1 | Rabbit | 1:1,000 | Thermo Fisher Scientific, MA, USA | 61-7300 |
| Occludin | Rabbit | 1:1,000 | Abcam, Cambridge, UK | ab31721 |
| GAPDH | Rabbit | 1:2,000 | Cell Signaling Technology, MA, USA | 2118 |

^1^ZO-1 = zonula occludens-1; GAPDH = glyceraldehyde-3-phosphate dehydrogenase.
